# Combination Therapy Strategies Against Multiple-Resistant *Streptococcus Suis*

**DOI:** 10.3389/fphar.2018.00489

**Published:** 2018-05-15

**Authors:** Yang Yu, Jin-Tao Fang, Mei Zheng, Qing Zhang, Timothy R. Walsh, Xiao-Ping Liao, Jian Sun, Ya-Hong Liu

**Affiliations:** ^1^National Risk Assessment Laboratory for Antimicrobial Resistance of Animal Original Bacteria, South China Agricultural University, Guangzhou, China; ^2^Guangdong Provincial Key Laboratory of Veterinary Pharmaceutics Development and Safety Evaluation, South China Agricultural University, Guangzhou, China; ^3^National Reference Laboratory of Veterinary Drug Residues, College of Veterinary Medicine, South China Agricultural University, Guangzhou, China; ^4^Department of Medical, Microbiology and Infectious Disease, Institute of Infection & Immunity, Heath Park Hospital, Cardiff, United Kingdom

**Keywords:** *Streptococcus suis*, combination therapy, checkerboard methods, *in-vivo* mouse model, multiple resistance

## Abstract

*Streptococcus suis* is a major swine pathogen, an emerging zoonotic agent responsible for meningitis, endocarditis and septicaemia followed by deafness in humans. The development of antimicrobial resistance in *S. suis* increases the risk for therapeutic failure in both animals and humans. In this study, we report the synergism of combination therapy against multi-resistant *S. suis* isolates from swine. Twelve antibiotic profiles were determined against 11 *S. suis* strains. To investigate their synergistic/antagonistic activity, checkerboard assay was performed for all the possible combinations. *In-vitro* killing curves and *in-vivo* treatment trials were used to confirm the synergistic activity of special combinations against *S. suis* dominant clones. In this study, 11 *S. suis* isolates were highly resistant to erythromycin, clindamycin, trimethoprim/sulfamethoxazole, and tetracycline with ratios of 80–100%, and the resistance percentages to enrofloxacin, florfenicol, and spectinomycin were ~50%. The checkerboard data identified two combination regimens, ampicillin plus apramycin and tiamulin plus spectinomycin which gave the greatest level of synergism against the *S. suis* strains. *In-vitro* kill-curves showed a bacterial reduction of over 3-logCFU with the use of combination treatments, whilst the application of mono-therapies achieve less than a 2-logCFU cell killing. *In-vivo* models confirm that administration of these two combinations significantly reduced the number of bacterial cells after 24 h of treatment. In conclusions, the combinations of ampicillin plus apramycin and tiamulin plus spectinomycin showed the greatest synergism and may be potential strategies for treatment of multi-resistant *S. suis* in animal.

## Introduction

*Streptococcus Suis*, a facultatively anaerobic Gram-positive coccus, is an important swine pathogen worldwide and causes considerable economic losses within the swine industry (Haas and Grenier, 2017). *S. suis* is also known as an emerging zoonotic agent since the first cases of human infection in 1986 (Perch et al., 1968). In swine, *S. suis* can cause meningitis, sepsis and arthritis, whereas the main clinical syndrome in humans is meningitis, followed by septicemia, pneumonia, endocarditis, arthritis, and septic shock (Gottschalk et al., 2007; Lun et al., 2007; Huong et al., 2014; Segura et al., 2017). *S. suis* can be a commensal or an opportunistic pathogen and invade pigs through the upper respiratory-, genital- and alimentary tracts (Nghia et al., 2011; Segura et al., 2016). Nevertheless humans infections usually occur via skin lesions (handling of or exposure to infected animals) or via the oral route (ingestion of contaminated pork-derived products) (Segura et al., 2016, 2017). Therefore, human disease mostly occurs occupationally in pig breeders, butchers, pork processing workers and veterinarians; and consumption of raw or uncooked pig products is another epidemiology factor of *S. suis* infections (Segura et al., 2017).

During the last 20 years, the number of human infections cases has been dramatically increased, including two outbreaks in China in 1998 and 2005 (Hu et al., 2000; Yu et al., 2006). There has also been a growth of morbidity in Thailand and Vietnam. On the contrary, in Western countries, almost all cases were sporadic and caused by occupational contact or exposure to pigs or swine products. Although the majority human cases were identified in Asian countries (Hui et al., 2005; Yu et al., 2006; Gottschalk et al., 2007; Nghia et al., 2011), *S. suis* infections are reported worldwide, including the United State (Fittipaldi et al., 2009; Dejace et al., 2017), Australia (Kennedy et al., 2008), Canada (Gottschalk et al., 2007; Gomez-Torres et al., 2017), New Zealand (Dickie et al., 1987), and most of Europe with a highly developed pig industry like The Netherlands, the United Kingdom (Hernandez-Garcia et al., 2017), France, and Spain (Goyette-Desjardins et al., 2014; Dutkiewicz et al., 2017). Between 2002 and 2013, a total of 1,642 *S. suis* infections were identified in 34 countries (Goyette-Desjardins et al., 2014); and in recent years new cases have been first reported occurring in many other countries, like Malaysia (Rajahram et al., 2017), Brazil (Doto et al., 2016), Togo (Prince-David et al., 2016), Japan (Taniyama et al., 2016), and India (Devi et al., 2017). However, it is noteworthy that the real morbidity of *S. suis* could be greatly underestimated due to misdiagnosis (Bojarska et al., 2016; Callejo et al., 2016).

Over the past decade, an increasing level of antibiotic resistance has been reported worldwide, which has been recognized as a global problem to public health (Hernandez-Garcia et al., 2017). Notwithstanding, the use of antimicrobial agents in animals is being strictly monitored, either as prophylactic, metaphylactic, or therapeutic use, in order to reduce the medium-long term risk of antimicrobial resistance in humans. Multidrug-resistant phenotypes of *S. suis* have been noted in both pigs and human cases. Penicillin and cephalosporins are the first choices against *S. suis* infection, however resistance to these two drugs was reported in Europe and China (Shneerson et al., 1980; Zhang et al., 2015). Tetracycline resistance in *S. suis* was found in North America, Asia, and many European countries ranging from 86.9% up to 100% in pigs (Seitz et al., 2016), and resistance in human meningitis cases was reported in Asia (Chu et al., 2009; Hoa et al., 2011). Extensive resistance has been reported against aminoglysides β-lactams, macrolide, lincosamide, trimethoprim, and amphenicols (Chu et al., 2009; Holden et al., 2009; Palmieri et al., 2011; Varela et al., 2013; Huang et al., 2015). Notably, the bacteria can be commensal or carried in various species, including birds, rabbits, cats, dogs, horses, cattle, fallow deer, and wild boars, which may cause widespread in nature and the persistence of endemic foci in pigs (Hernandez-Garcia et al., 2017). It is no doubt that *S. suis* is cause for increasing concern against the swine industry and human health.

In this work, we try to find prospects to treat multi-drug resistant *S. suis* by employing combination therapy. One or two antimicrobial agents per each category were chosen for this study, which were conventional and widely used in Chinese veterinary clinic for treating *S. suis* diseases. The resistant characteristics of 11 *S. suis* isolates from diseased pigs were tested for MIC as phenotype and confirmed by genotype using PCR and sequencing. Synergistic interactions between different antibiotics were investigated against the multiple-resistant *S. suis* infection via combination therapy. Then the synergism of certain combinations was further confirmed by *in-vitro* and *in-vivo* treatment trials.

## Materials and methods

### Bacteria and susceptibility tests

*S. suis* isolates were collected from diseased or dead pigs at an animal hospital in Foshan Guangzhou, China, between 2008 and 2013, and were cultured in Tryptical Soy broth or agar plate (Becton Dickinson Ltd., US) containing 5% defibrinated sheep blood or 5% new born calf serum (Ruite Bio-tec Company, Ltd., Guangzhou China) at 37°C. All isolates were identified as *S. suis* by MALDI-TOF/MS (Shimadzu-Biotech). Serotype was tested by slide agglutination test (Statens Serum Institut, Copenhagen, Denmark) and PCR test. Twelve conventional antibiotics, which were commonly used for treatment of *S. suis* infections in veterinary clinic, were tested in total for the minimal inhibitory concentrations (MICs), including ampicillin (AMP), ceftiofur (CEF), enrofloxacin (ENR), apramycin (AP), spectinomycin (SPT), tetracycline (TET), erythromycin (ERY), chloramphenicol (CHL), florfenicol (FFN), tiamulin (TIA), trimethoprim/sulfamethoxazole (T/S), and clindamycin (CLI). Broth micro-dilution method was performed in Mueller Hinton (MH) broth in triplicates regarding the guideline of Clinical and Laboratory Standards Institute (2013). To confirm the resistant phenotype, the antimicrobial resistant determinants especially for erythromycin, clindamycin, and tetracycline were detected by PCR amplification. In brief, PCR started from 5 min of initial denaturation at 95°C; followed by 30 cycles of denaturation, annealing and extension at 95, 50–62, and 72°C for 45 s, 45 s, and 60 s per kb, respectively; then finished with a final extension at 72°C for 7 min. The positive amplicons were selected randomly for sequencing analysis (The Beijing Genomics Institute, BGI). The primers and annealing temperatures were listed in Table S1. The ATCC 43765 was used as the susceptible control, and 11, 41, and 1025 were randomly selected for the following experiments referring the resistant characteristics.

### Drug interaction assay

The synergistic interaction of all possible combinations was investigated by checkerboard method using 96-well micro-dilution plates. *S. suis* strains were overnight cultured in MH broth, washed twice by normal saline and re-suspended in MH medium, then inoculated to the final cell density of 5 × 10^5^ CFU/mL. Antibiotics concentrations of 1/8, 1/4, 1/2, 1, 2, 4, and 8 × MIC of each compound were tested. The combined therapeutic MIC was defined as the concentration of no visible growth after 24 h incubation at 37°C. The fractional inhibitory concentrations index (FICI) for combination effect was calculated as following:

(1) $$\begin{array}{r} FICI=\frac{MIC\;of\;A\;in\;combination}{MIC\;of\;A\;alone}+\frac{MIC\;of\;B\;in\;combination}{MIC\;of\;B\;alone} \end{array}$$

from which synergism is defined as FICI ≤ 0.5; indifference was indicated by a 0.5 < FICI ≤ 4; and a value of >4 was defined as antagonistic (Louie et al., 2011).

### Killing curves of synergistic combinations

Following overnight incubation at 37°C, bacterial suspension was diluted to 10^6^ CFU/ml as an initial inoculum using normal saline. Working system was composed of a drug-free control, mono-therapy of drug A and B, and the combination of A and B together. Concentrations of 1 × MIC and 2 × MIC for every organism were tested here. Then, 100 μl suspension was sampled and appropriately diluted for bacterial counting at 0, 3, 6, 9, and 12 h, respectively. Only synergistic combination was estimated for killing activity against four *S. suis* strains. A minimum of two independent experimental runs was performed.

### Preliminary combination therapeutic verification *in vivo*

The *in-vivo* studies were performed on mouse thigh models with neutropenia. Specific-pathogen-free (SPF) female ICR mice, aging six-week-old and weighing 25 ± 2 g, were administrated with cyclophosphamide (Yuanye Biotechnology Co., Ltd, Shanghai, China) for inducing Neutropenia (neutrophils ≤ 100/mm^3^). An initial dose of 150 mg/kg of cyclophosphamide was injected intraperitoneally over the first 4 days and followed by a single dose of 100 mg/kg on the fifth day, prior to thigh infection.

Then the neutropenic mice were infected with a 100 μl intramuscular injection of exponentially growing bacterial suspension (10^7^ CFU/mL) into each posterior thigh muscle. *In-vivo* treatments with antibiotics or placebo were initiated at 2 h following the bacterial inoculation. Therapeutic dosages of each drug were derived from a two-fold MIC value against four *S. suis* strains, respectively. In each trial, 16 mice were randomly divided into four groups and received single administration accordingly, with PBS as control, drug A as mono-therapy, drug B as mono-therapy, and A and B together as combination regimen. Following 24 h of treatment, groups of 4 mice were sacrificed and thigh homogenates (8 thigh infections) were sampled for bacterial burden quantification.

### Ethics statement

The SPF female ICR mice were purchased from Hunan Silaikejingda Lab Animal Ltd., Hunan, China. Groups of four mice were breeding in SPF environment with a half-half of light-dark circle in accordance with National Standards for Laboratory Animals of China (GB 14925–2010). The *in-vivo* mouse studies were approved by the Guangdong Association for Science and Technology [ID: SYXK (Guangdong) 2014–0136] and the Animal Research Committees of SCAU. All the protocols were followed the Guangdong Laboratory Animal Welfare and Ethics guidelines and the Institutional Animal Care and Use Committee guidelines of the South China Agricultural University.

## Results

### Resistant profiles

**Figure 1:**
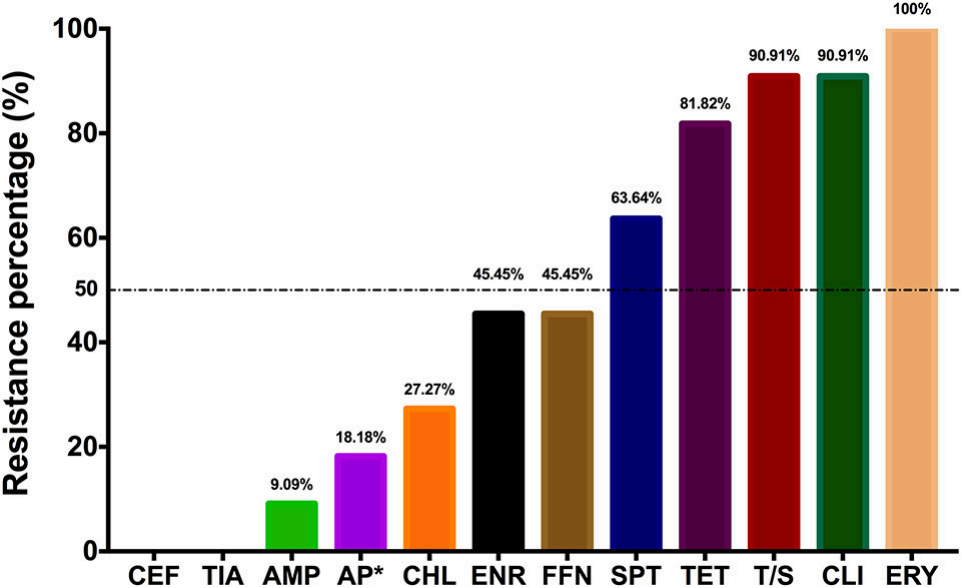
Bar chart shows the resistance ratio of *S. suis* isolates to 12 antimicrobial agents. AMP, Ampicillin; AP, Apramycin; CEF, Ceftiofur; CHL, Chloramphenicol; CLI, Clindamycin; ENR, Enrofloxacin; ERY, Erythromycin; FFN, Florfenicol; SPT, spectinomycin; S/T, Compound Sulfamethoxazole; TET, Tetracycline; TIA Tiamulin. ^*^Two isolates with apramycin MIC of 256 μg/ml were deemed as resistant strains, as no applicable breakpoint for apramycin.

Eight isolates were identified as *S. suis* serotype 2 strains (SS2), but the other three were non-typable (Table S2). The MICs distribution of eleven isolates were shown in Table 1, where the MIC_50_ and MIC_90_ were list as well. In total eleven isolates were highly resistant to spectinomycin, tetracycline, trimethoprim/sulfamethoxazole, clindamycin, and erythromycin with drug resistant percentage of 50% or higher (Figure 1), referring to the resistance breakpoints (Michigan State University DCPAH, 2014); but susceptible to ampicillin, ceftiofur, and tiamulin still. Notably *S. suis* 1025, 11 and 41 appeared to be multiple resistant to more than seven different antibiotics (Table S2). On the other hand, the resistant genes of *tetM, lnuB, and erm(B)* were highly distributed in these *S. suis* isolates with the detection rates of 72.73, 54.55, and 36.36%, respectively (Table S3). *S. suis* 1025, 11 and 41 were found harboring *erm(B), tetM, tetO, tetL, and lnuB* genes at the same time, which confirmed the co-resistance to erythromycin, tetracycline and clindamycin (Table 1, Tables S2, S3, and Figure S1). Another methylase gene *erm(A)* and aminoglycoside-resistant gene *aph3*′ were found in isolates 41 and 11. The genotypes inferred from PCR amplification were excellently matched the resistant phenotypes as tested by MIC.

**Table 1:**
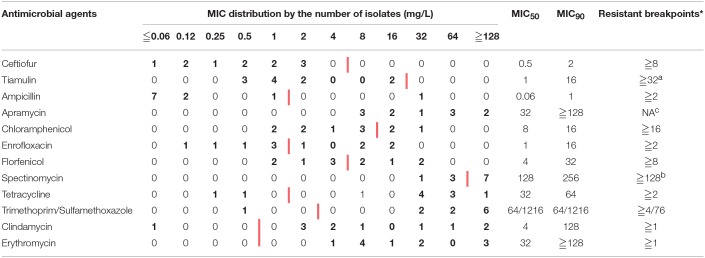
Distribution of minimum inhibitory concentrations in 11 *Streptococcus suis isolates*.

^*^Resistant breakpoint data is provided referring to the Resistance Breakpoints for Antimicrobials Used in Animals from Diagnostic Center for Population and Animal Health, Michigan State University, which is summarised from the CLSI documents Performance Standards for Antimicrobial Susceptibility Testing (M100-S24) and (VET01-S2).

*^a^For Actinocacillus pleuropneumoniae; ^b^For bovine respiratory disease; ^c^NA, not applicable; Red vertical line for breakpoints. Bold value represents the number of strains*.

### Synergism assay

Based on checkerboard data, an independent interaction (FICI, ranging from 1 to 3) was shown by most of the possible combinations against *S. suis* ATCC 43765 (Table 2). Two combination regimens of AMP plus AP and TIA plus SPT scored an FICI of 0.5 and exhibited synergistic antibacterial effectiveness. Similar results were found in a further test of these two synergistic combinations against *S. suis 1025, 11, and 41* isolates. It was notable that combination administration of these two groups helped decrease antimicrobial susceptibility of both drugs. As shown in Table 3, when AMP and AP were used together, MICs of combined therapy were two- to four-fold lower than AMP used alone, and vice versa AP MICs also decreased two- to four-fold together with AMP. Similarly, in TIA/SPT group, organisms were four times more sensitive to TIA in the presence of SPT, notwithstanding that they were basically resistant to SPT (Table 1). On the other way, SPT MICs in combination with TIA fell back into the intermediate zone against all three pathogens. Interestingly, the FICI values of both groups against *S. suis* 1025 were somehow a little higher than the definition of synergy action, as well as AMP/AP against *S. suis* 41.

**Table 2:** *In vitro* interactions of 12 antibiotics against *S. suis* ATCC 43765 calculated by checkerboard method.

**Checkerboard**	**AP**	**CHL**	**ENR**	**FFN**	**TIA**	**ERY**	**CLI**	**AMP**	**TET**	**S/T**	**CEF**	**SPT**
Apramycin		I	I	I	I	I	I	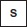	I	I	I	I
Chloramphenicol	2		I	I	I	I	I	I	I	I	I	I
Enrofloxacin	1.5/2	2		I	I	I	I	I	I	I	I	I
Florfenicol	2	1.5	2		I	I	I	I	I	I	I	I
Tiamulin	2	2	2	2		I	I	I	I	I	I	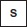
Erythromycin	2	2	2	2	0.75/1		I	I	I	I	I	I
Clindamycin	1/2	2	2	2	2	2		I	I	I	I	I
Ampicillin	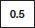	2	0.75	2/3	2	2	1.5/2		I	I	I	I
Tetracycline	2	2	2	2	1.5/2	1.5/2	2	2		I	I	I
Trimethoprim/Sulfamethoxazole	1.5/2.5	2	1/2	1.5/2	2	2	2	2	2/3		I	I
Ceftiofur	1/2	2	0.75/1	2	1.5/2	1.5/2	2	1/1.5	2	1.5/2		I
Spectinomycin	2	2	1.5	2	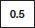	2	2	2	2	2	2	

*FICI, Fractional inhibitory concentration index; S, Synergistic (FICI ≤ 0.5); I, independent (0.5 < FICI ≤ 4); A, Antagonistic (FICI>4)*.

**Table 3:** Combination MICs and FICI values of AMP/AP and SPT/TIA against *S. suis* ATCC 43765, 1025, 41, and 11 strains, determined from checkerboard tests.

**Strain**	**MIC μg/mL**				**FICI**	**MIC μg/mL**				**FICI**
	**AMP**	**AP**	**AMP combined with AP**	**AP combined with AMP**		**SPT**	**TIA**	**SPT combined with TIA**	**TIA combined with SPT**	
ATCC 43765	0.03	32	0.0075	8	0.5	32	1	8	0.25	0.5
1025	32	256	16	64	0.75	128	16	64	4	0.75
41	0.0625	8	0.031	4	1	256	0.5	64	0.125	0.5
11	0.125	8	0.031	2	0.5	256	16	64	4	0.5

### *In-vitro* synergism of AMP/AP and TIA/SPT

**Figure 2:**
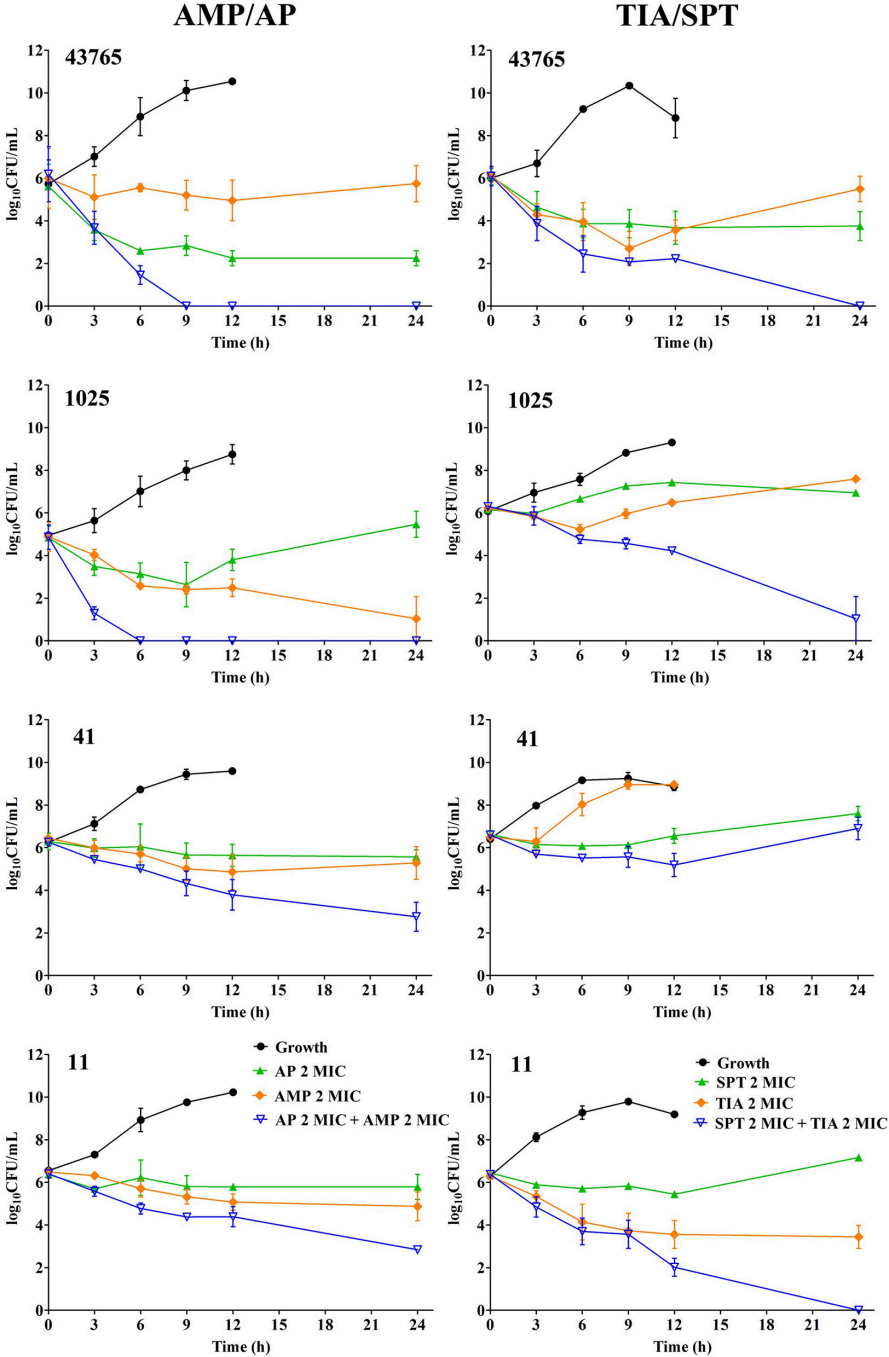
*In-vitro* time-killing curves of synergistic combination against four *S. suis* strains including ATCC 43765, 1025, 41 and 11. Symbols represent means and error bars are standard deviations (*n* = 3).

In Figure 2, killing activity of antibiotics alone was very limited even against the *S. suis* ATCC 43765. However bacterial reductions of 5–6 logCFU were observed in combined treatment with AMP/AP and TIA/SPT against isolate 1025. Similar outcomes were found in groups of TIA/SPT against *S. suis* 11. But when dealing with *S. suis* 41, only bacteriostatic effect was observed regardless of the drug combination. Notably, after bacterial suspension reaching the plateau stage, a downward trend was observed in all the control arms (Because of which, plots at 24 h of the control arms were not shown).

### *In-vivo* therapy verification

**Figure 3:**
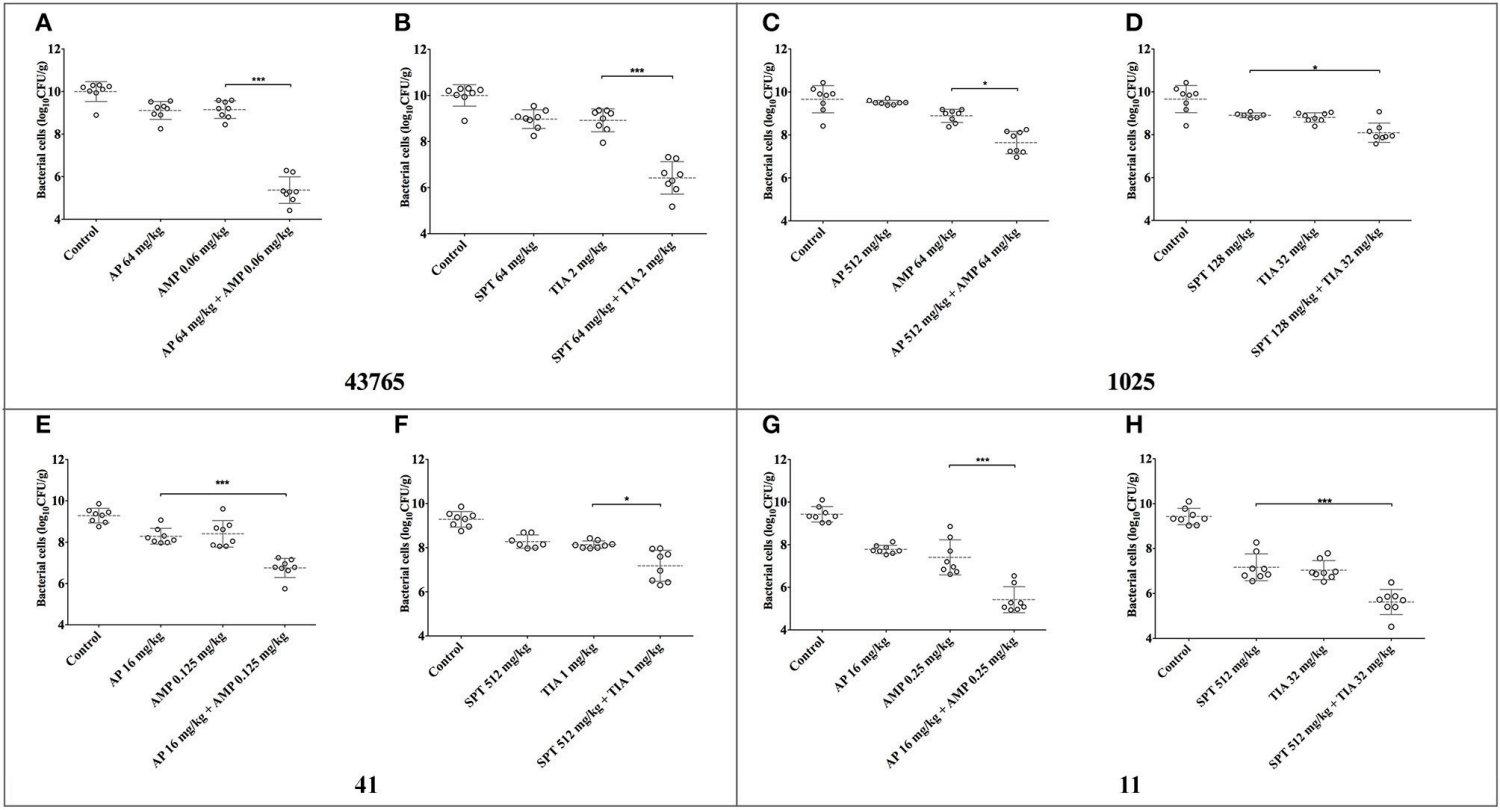
Bacteria density in mouse thigh muscles (logCFU/g) after 24 h of monotherapy or combination therapy using different synergistic regimens against four *S. suis* strains. Combinations of ampicillin plus apramycin and tiamulin plus spectinomycin were tested against four *S. suis* isolates. Significant difference was shown as ^*^ and ^***^ representing *P* < 0.05 and *P* < 0.001, respectively. AMP, ampicillin; AP, apramycin; TIA, tiamulin; SPT, spectinomycin. **(A,B)** Show the data for ATCC 43765; **(C,D)** for *S. suis* 1025; **(E,F)** for *S. suis* 41; **(G,H)** for *S. suis* 11.

Data of *in-vivo* synergistic treatment are displayed in Figure 3. Groups of combination therapy generated significant bacterial killing (*Two-tail t-test*; dataset comparisons with differences of *P*-values of <0.05 were considered to be statistically significant).

Significant synergism of both combinations was observed when treating against reference strain ATCC 43765 (*P* < 0.001). As expected, wild type strains showed stronger viability than the reference strain, even though reproduction of bacteria had been inhibited following combination therapy. AP of 512 mg/kg plus AMP of 64 mg/kg restrained the growth of *S. suis* 1025 to ~2-logCFU/g, which was lower than that of mono-therapy at 24 h (*P* < 0.05). Similarly, AP/AMP combined treatment was effective against the infections induced by the other two pathogens even when administrated with lower doses (*P* < 0.001). Following administration of TIA plus SPT, there was a remarkable inhibition of bacterial growth observed in the mouse thighs infected with *S. suis* 41 and 11 (*P* < 0.05 and *P* < 0.001, respectively). However, significant difference of bacterial density was observed only between the assembly of SPT alone and that of SPT/TIA against *S. suis* 1025 (*P* < 0.05).

## Discussion

In this study, 8 of 11 isolates were identified as serotype 2 in the percentage of 72.73%, which suggested that in the Foshan area serotype 2 might be the most popular serotype in *S. suis*. In addition, *S. suis* serotype 2 was mainly responsible for those two large outbreaks in China in 1998 and 2005 (Hu et al., 2000; Yu et al., 2006). So far, over 20 countries and ~700 human cases have been reported, which were caused by the highly virulent serotype 2 *S. suis* (Mai et al., 2008; Holden et al., 2009). In humans, it causes severe health problems including meningitis and septicemia shock, which may prolong the treatment. Certainly, *S. suis* serotype 2 strains are the most prevalent responsible for both swine and human diseases, among the total 35 serotypes that have been described during the last decades.

Increasing attention has been paid to *S. suis*, not only because of its role in severe infectious cases in humans, but also due to its involvement in antimicrobial resistance caused by the abuse of antibiotics (Palmieri et al., 2011; Varela et al., 2013). In this study, *S. suis* isolates were resistant to erythromycin, clindamycin, trimethoprim/sulfamethoxazole, and tetracycline with resistant percentage over 80% to 100%, and showed decreasing susceptibilities to enrofloxacin, florfenicol, and spectinomycin with resistant frequency ~50% (Figure 1). Besides, high MICs of apramycin were observed despite there is no definition of apramycin breakpoint neither from CLSI nor EUCAST. Although all the isolates were resistant to erythromycin, extremely higher MICs of over 256 mg/L were observed in *S. suis* 1025, 11, and 41 strains and followed by MIC of 64 mg/L in isolates 114 and NJ-5 (Table S2). As a predominant resistant mechanism, the presence of ribosomal methylase genes *ermA* and/or *ermB* might be the reason causing highly resistant phenotype to erythromycin (Table S3), and, less frequently, harboring the efflux pump genes *mefA* and *msrD* (only in isolate 114) could mediate a medium level resistant to erythromycin. Although there are many genes mediating tetracycline resistance, the ribosomal protection genes of *tetM* and *tetO* were notably detectable in *S. suis* and a reduced frequency to efflux gene *tetL* (Roberts, 2005; Huang et al., 2015). In agreement with previous reports (Zhang et al., 2015), highly prevalence of *tetM* (8 of 11 isolates) and followed by *tetO* (three isolates) were detected in this study. However, detection of efflux gene *tetL* were also observed in *S. suis* 1025, 11, and 41 strains. The *lnuA* and *lnuB* gene are members of *lnu* gene family encoding lincosamide nucleotidyltransferase enzymes, then further causing the resistant to lincosamides (Montilla et al., 2014). Six from 11 isolates were detected with *lnuB* gene, among which two isolates were also harboring the *lnuA* gene. Isolates, 2015, 11, and 41, were identified as multiple resistant *S. suis* strains based on both phenotype of the MIC value and genotype of co-existing of different resistant genes. For these three, antimicrobial resistant genes of *ermB, tetM, tetO, tetL*, and *lnuB* were co-existing, and the 11 and 41 were also carrying the *aph3*′ gene mediating aminoglycosides resistance. It is revealed that resistant genes to erythromycin, tetracycline, and clindamycin were highly prevalent in *S. suis* isolates singly or in combination with each other.

By the way, chloramphenicol MICs over 16 mg/L were observed against *S. suis* 1025, 11, and 41. However, no detection of *cfr* gene (conferring resistance phenicols, lincosamides, oxazolidinones, pleuromutilins, and streptogramin) were observed in these strains, inconsistently with previous report that *cfr* gene was found in *S. suis* isolates from porcine in China (Wang et al., 2013). However, the resistance to chloramphenicol might be caused by other mechanisms, for example, by *cat* genes encoding chloramphenicol acetyltransferase; or genetic mutation. Similar results were also observed in MICs of spectinomycin, of which most isolates are resistant, but lack of the presence of resistant genes. Interestingly, high value of spectinomycin MICs were found in isolates 11 and 41, however, as in the same group with spectinomycin, apramycin MICs against those strains were observed oppositely in a quite low level. In this study, only few groups of antimicrobial resistant genes were detected, which might be not sufficient to explain the all the resistant phenotypes. Besides, under the antimicrobial selection pressure, excepting for acquiring resistant genes, *S. suis* can also survive by other resistant mechanisms, like up-regulating the express of efflux pump, developing tolerance to antibiotics, or forming biofilms.

In brief, *S. suis* can be an abundant antibiotic resistance reservoir contributing to the spread of resistance genes between animal and human (Palmieri et al., 2011), and it is important to introduce an effective strategy to treat multiple resistant *S. suis*. In this work, 12 antimicrobial agents were tested for synergism using checkerboard trials, and surprisingly two combinations of ampicillin plus apramycin and spectinomycin plus tiamulin emerged to be synergistically effective (FICI of 0.5) against *S. suis* ATCC43765 (Table 2). A floating FICI value was observed from the following checkerboard tests against the clinical isolates 1025, 11 and 41. A FICI of 0.75 for ampicillin plus apramycin against 1025 may be due to MICs of 1025 to ampicillin and apramycin were extremely higher than other strains, and 1025 is the only isolates which resistant to ampicillin (Table S2). However, MICs cannot explain the higher FICIs of 0.75–1 in combination of ampicillin plus apramycin against strain 41 and spectinomycin plus tiamulin against 1025, respectively. The synergism was investigated by *in-vitro* dynamic killing curves (Figure 2) as well, from which the conspicuous synergistic killing activity (more than 3-logCFU/ml reduction) was observed in both combined strategies. Exceptionally, neither tiamulin and spectinomycin alone nor combined together could inhibit the growth of *S. suis* 41 *in-vitro*. On the other hand, interestingly, decreased cell density was observed in all the growth curves after bacterial density began reaching stable status in broth medium, which may be due to nutritional deficiency and large population density.

Further confirmation on the antimicrobial activity of these two combination therapies were carried on *in-vivo* mouse model. Before treatment, these four strains were colonizing well in the mouse thigh as expected. In Figure 3, bacterial cells multiplied to ~10-log_10_CFU/g at 24 h after inoculation, which was more viable and virulent than the *in-vitro* broth medium. The combined-therapeutic effectiveness was significantly (*P* < 0.05, or *P* < 0.001) more superior than mono-therapy for both pairs of combination for the four *S. suis* strains. Differences of 2–5 logCFU/g were achieved between control groups and the combined treatment groups, and differences of 2–4 logCFU/g were observed between mono-administration and the combination therapies. Nonetheless only 1-logCFU/g drop of bacterial counts was shown in spectinomycin combined tiamulin, which was still significantly decreased than monotherapy against isolate 1025 (*P* < 0.5).

However, we noticed there were some shorts for this study. First, only three multiple-resistant *S. suis* has been detected and tested for combination therapies, which might be insufficient to comprehensively understand the effectiveness of combined therapy. In addition, preliminary guidance for rational combination therapy was provided by synergy studies in mice, which may exert optimum microbiological outcomes. Nevertheless, since the complicated *in-vivo* condition, like dynamic drug concentrations in target tissue, bacterial virulence and host immune response in different species, cannot be completely mimicked by mouse model. Outcomes from mouse model may not be applied in the clinic directly. For instance, the dosages used in mouse trials were simply extrapolated from two-fold from the MIC value against each pathogen, which were not applicable for clinical treatment. Besides, it is a single administration used for *in-vivo* treatment in mouse models during a 24-h treatment. For the combination of ampicillin plus apramycin, ampicillin is belonging to a time-dependent beta-lactams antibiotic with none or short post-antimicrobial effects, and apramycin belongs to aminoglycosides which is concentration dependent. Based on these different pharmacokinetic and pharmacodynamic characteristics, it is recommended that ampicillin might be administrated multiple times daily in contrast to a single dosing of apramycin. As to tiamulin combined with spectinomycin, AUC/MIC is the most reasonable parameter available to the pharmacokinetics and pharmacodynamics profile (Craig, 1998; Xiao et al., 2016), therefore single daily dosages might be appropriate. However, previous report reveals that valnemulin, another pleuromutilin antibiotic, has shown a better efficacy with spilt dosages than single administration (Zhao et al., 2014).

Synergism of ampicillin plus apramycin is probably due to the increased permeation of aminoglycosides into cytoplasm after beta-lactams inhibiting the cell-wall synthesize by binding with penicillin binding proteins (PBPs). According to this hypothesis, penicillin has the ability to lower the MICs of aminoglycosides, and in which case penicillin may be administrated prior to aminoglycosides (Costa and Botta, 1984). Interestingly, we also found a decrease in the value of ampicillin MICs combined with apramycin (Table 3), however the mechanism remains unclear. Reports on the synergism of tiamulin combined with spectinomycin are even more limited. It seems like both of them are acting on bacterial ribosome and blocking the synthesis of the 30S protein. Combination therapy of tiamulin plus spectinomycin may be an appealing option for retaining the clinical utility of spectinomycin against such resistant strains.

“Maximize therapeutic efficacy while minimizing bacterial resistance” is the main goal of using of antimicrobials in both clinical and agriculture (Varela et al., 2013). In clinical, timely and prudent use of antimicrobial agents may be the best shot to reduce the impact and spread of antimicrobial-resistant pathogens for treating of *S. suis* infection in human. On the contrary, due to the consideration of public health, not many choices are left for veterinary *S. suis* diseases especially for resistant organisms. Strategies to cut down the impact of *S. suis* diseases and the dissemination of antimicrobial resistance should be focused on improving the management and environment of swine farming, together with strategic medication of clinically diseased animals (Varela et al., 2013). In current study, it was focused on the veterinary antimicrobial agent only. However, considering *S. suis* is an opportunistic and zoonotic pathogen, further study and verification of combining p0enicillins with aminoglycosides should be studied especially for human antimicrobial agents. A combination of pleuromutilin plus aminoglycosides may only be applicable to veterinary use, since retapamulin is the only pleuromutilin agent approved by the FDA for external use against *Staphylococcus aureus* infection in human clinics.

In summary, we found two combinations, ampicillin plus apramycin and tiamulin plus spectinomycin, both offering a substantially strengthened and more synergistic antibacterial activity against multi-resistant *S. suis* strains from porcine. Hopefully, our findings might also shine light on alternative combination therapies for human infection caused by porcine streptococcosis.

## Author contributions

Y-HL and YY conceived of this study and designed the experiment; YY drafted the manuscript; J-TF, MZ, and QZ carried out the *in-vitro* Experiments and analyzed the related data; X-PL and JS performed the *in-vivo* mouse model and analyzed the related data; TW revised the manuscript. All authors read and approved the final manuscript.

### Conflict of interest statement

The authors declare that the research was conducted in the absence of any commercial or financial relationships that could be construed as a potential conflict of interest. The reviewer PF and handling Editor declared their shared affiliation.
