# Supplementary material for: Combination Therapy Strategies Against Multiple-Resistant Streptococcus Suis
Source: Front Pharmacol. 2018 May 15;9:489. doi: 10.3389/fphar.2018.00489 (PMC5962770; doi:10.3389/fphar.2018.00489)
Supplement: Supplementary file 1 [file Table_1.docx]

**Combination Therapy Strategies against Multiple-Resistant *Streptococcus Suis***

Yang Yu^1,2,3,4^, Jin-Tao Fang^1,2,3^, Mei Zheng^3^, Qing Zhang^3^, Timothy R Walsh^4^, Xiao-Ping Liao^1,2,3^, Jian Sun^1,2,3^, Ya-Hong Liu^1,2, *^

^1^ National Risk Assessment Laboratory for Antimicrobial Resistance of Animal Original Bacteria, South China Agricultural University, Guangzhou, 510642, China

^2^ Guangdong Provincial Key Laboratory of Veterinary Pharmaceutics Development and Safety Evaluation, South China Agricultural University, Guangzhou, China

^3^ College of Veterinary Medicine, National Reference Laboratory of Veterinary Drug Residues, South China Agricultural University, Guangzhou, China

^4^ Department of Medical, Microbiology and Infectious Disease, Institute of Infection & Immunity, Heath Park Hospital, Cardiff, UK

**Running head:** Synergism of combination therapy against *S. suis*

**Keywords:** *Streptococcus suis*, combination therapy, checkerboard methods, *in-vivo* mouse model, multiple resistance

***Correspondence author:**

Ya-Hong Liu, Veterinary Pharmacology Department, College of Veterinary Medicine, South China Agricultural University, Guangzhou, P.R. China.

E-mail : [lyh@scau.edu.cn](mailto:lyh@scau.edu.cn). Tel : +86-020-85280006

**Table S1.** The sequence of primers used for PCR amplification of serotypes and antimicrobial resistant gene (ARG).

| **Gene** | **Sequence (5’-3’)** | **Size (bp)** | **Annealing Temperature** | **Reference** |
| --- | --- | --- | --- | --- |
| **Serotype** |  |  |  |  |
| *cps2-F* | GTTGAGTCCTTATACACCTGTT | 461 | 60°C | (Wisselink et al., 1999) |
| *cps2-R* | CAGAAAATTCATATTGTCCACC |  |  |  |
| **MLS_B_ ARGs** |  |  |  |  |
| *erm(A)-F* | GTTCAAGAACAATCAATACAGA G | 421 | 54°C | This study |
| *erm(A)-R* | GGATCAGGAAAAGGACATTTTAC |  |  |  |
| *erm(B)-F* | GAAAAGGTACTCAACCAAATA | 639 | 51°C | This study |
| *erm(B)-R* | AGTAACGGTACTTAAATTGTTTA C |  |  |  |
| **Macrolide ARGs** |  |  |  |  |
| *mphA-F* | AACTGTACGCACTTGC | 837 | 50°C | (Tait-Kamradt et al., 2000) |
| *mphA-R* | AACTGTACGCACTTGC |  |  |  |
| *mefA-F* | AGTATCATTAATCACTAGTGC | 346 | 51°C | This study |
| *mefA-R* | TTCTTCTGGTACTAAAAGTGG |  |  |  |
| *msrA-F* | GCAAATGGTGTAGGTAAGACAACT | 399 | 57.5°C | (Sutcliffe et al., 1996) |
| *msrA-R* | TAAAACAAATGTAGTGTACTA |  |  |  |
| *msrD-F* | CCTTATCGGCACAGGTTCAT | 500 | 57°C | (Lüthje and Schwarz, 2007) |
| *msrD-R* | GCCTTCCGGAGCTCCTACTT |  |  |  |
| **Tetracycline ARGs** |  |  |  |  |
| *tetM-F* | GTGGACAAAGGTACAACGAG | 406 | 56°C | (Warsa et al., 1996) |
| *tetM-R* | CGGTAAAGTTCGTCACACAC |  |  |  |
| *tetO-F* | ATTTGCCAATGGTATATCAAG | 475 | 51.4°C | This study |
| *tetO-R* | AATATAATTCACCGTTTGTCG |  |  |  |
| *tetK-F* | GATCAATTGTAGCTTTAGGTGAAGG | 155 | 62°C | (Malhotra-Kumar et al., 2005) |
| *tetK-R* | TTTTGTTGATTTACCAGGTACCATT |  |  |  |
| *tetL-F* | TCGTTAGCGTGCTGTCATTC | 738 | 52°C | This study |
| *tetL-R* | GTATCCCACCAATGTAGCCG |  |  |  |
| **Lincomycin ARGs** |  |  |  |  |
| *lnuA-F* | GGTGGCTGGGGGTAGTATTAACTGG | 323 | 57°C | (Lina et al., 1999) |
| *lnuA-R* | GCTTCTTTTGAAATACATGGTATTTTTCGA |  |  |  |
| *lnuB-F* | CCTACCTATTGTTTGTGGAA | 906 | 54 | (Bozdogan et al., 1999) |
| *lnuB-R* | ATAACGTTTACTCTCCTATTC |  |  |  |
| *lnuC-F* | AATTTGCAATAGATGCGGA GA | 1100 | 50°C | (Lüthje and Schwarz, 2007) |
| *lnuC-R* | TCATGTGCATTTTCAT CA |  |  |  |
| **Aminoglycoside ARG** |  |  |  |  |
| *aph3'-F* | TATAGAAAGTCTACGAGG | 582 | 55°C | This study |
| *aph3'-R* | TATAGAAAGTCTACGAGG |  |  |  |
| **Phenicol ARGs** |  |  |  |  |
| *cfr-F* | TAAGAAGTAATAATGAGC | 518 | 58°C | This study |
| *cfr-R* | TATAGAAAGTCTACGAGG |  |  |  |
| *fexA-F* | TTGGGAAGAATGGTTCAGGG | 977 | 60.5°C | This study |
| *fexA-R* | ATCGGCTCAGTAGCATCACG |  |  |  |

**Table S2.** Minimal inhibitory concentration of 12 antibiotics and serotype for 11 *S. suis* isolates.

| **NO.** | **Serotype** | **MIC (mg/L)** | | | | | | | | | | | |
| --- | --- | --- | --- | --- | --- | --- | --- | --- | --- | --- | --- | --- | --- |
|  |  | **AMP** | **AP** | **CEF** | **CHL** | **CLI** | **ENR** | **ERY** | **FFN** | **SPT** | **T/S** | **TET** | **TIA** |
| **11** | 2 | 0.125 | 8 | 0.5 | **32** | **256** | **16** | **>256** | **32** | **256** | **>****64/1216** | **64** | 16 |
| 12 | 2 | 0.0625 | 64 | 2 | 2 | 2 | 8 | 8 | 16 | 128 | >64/1216 | 256 | 2 |
| 40 | 2 | 0.015 | 16 | 1 | 2 | 4 | 0.5 | 32 | 2 | 64 | >64/1216 | 8 | 1 |
| **41** | 2 | 0.0625 | 8 | 0.125 | **16** | **128** | **16** | **>256** | 1 | **256** | **64/1216** | **128** | 0.5 |
| 75 | NT | 0.125 | 64 | 0.25 | 8 | 2 | 1 | 8 | 8 | 128 | >64/1216 | 32 | 1 |
| 94 | 2 | 0.0625 | 64 | 0.5 | 8 | 8 | 1 | 8 | 4 | 128 | >64/1216 | 32 | 1 |
| 114 | 2 | 0.03 | 8 | 1 | 1 | 32 | 0.25 | 64 | 4 | 64 | 32/608 | 32 | 0.5 |
| **1025** | 2 | **32** | **256** | 2 | **16** | **64** | **8** | **>256** | **32** | **128** | **>64/1216** | **128** | 16 |
| 1-2 | NT | 1 | 256 | 0.125 | 8 | 4 | 2 | 8 | 8 | 256 | 64/1216 | 0.25 | 0.5 |
| NJ-5 | NT | 0.03 | 16 | 2 | 4 | 2 | 0.125 | 64 | 4 | 64 | 32/608 | 32 | 2 |
| 43765 | 2 | 0.03 | 32 | 0.0075 | 1 | 0.015 | 1 | 4 | 1 | 32 | 0.5/9.5 | 0.5 | 1 |

AMP: Ampcillin; AP: Apramycin; CEF: Ceftiofur; CHL: Chloramphenicol; CLI: Clindamycin; ENR: Enrofloxacin; ERY: Erythromycin; FFN: Florfenicol; SPT: spectinomycin; T/S: Trimethoprim/Sulfamethoxazole; TET: Tetracycline; TIA: Tiamulin.

NT: Non-typable.

**Table S3.** Resistance genes in *S. suis* strains.

| **Resistance gene** | ***1025*** | ***41*** | ***11*** | 12 | 40 | 75 | 94 | 114 | 1-2 | NJ-5 | 43765 | Prevalence (%) |
| --- | --- | --- | --- | --- | --- | --- | --- | --- | --- | --- | --- | --- |
| *erm(A)* | - | + | + | - | - | - | - | - | - | - | - | 18.18 |
| *erm(B)* | + | + | + | - | - | - | - | + | - | - | - | 36.36 |
| *mphA* | - | - | - | - | - | - | - | - | - | - | - | 0 |
| *mefA* | - | - | - | - | - | - | - | + | - | + | - | 18.18 |
| *msrA* | - | - | - | - | - | - | - | - | - | - | - | 0 |
| *msrD* | - | - | - | - | - | - | - | + | - | - | - | 9.09 |
| *tetM* | + | + | + | + | + | - | + | + | - | + | - | 72.73 |
| *tetO* | + | + | + | - | - | - | - | - | - | - | - | 27.27 |
| *tetK* | - | - | - | - | - | - | - | - | - | - | - | 0 |
| *tetL* | + | + | + | - | - | - | - | - | - | - | - | 27.27 |
| *lnuA* | + | + | - | - | - | - | - | - | - | - | - | 18.18 |
| *lnuB* | + | + | + | - | + | - | - | + | - | + | - | 54.55 |
| *lnuC* | - | - | - | - | - | - | - | - | - | - | - | 0 |
| *aph3’* | - | + | + | - | - | - | - | + | - | - | - | 27.27 |
| *cfr* | - | - | - | - | - | - | - | - | - | - | - | 0 |
| *fexA* | - | - | - | - | - | - | - | - | - | - | - | 0 |

**Figure S1. PCR gel electrophoresis of resistant genes**


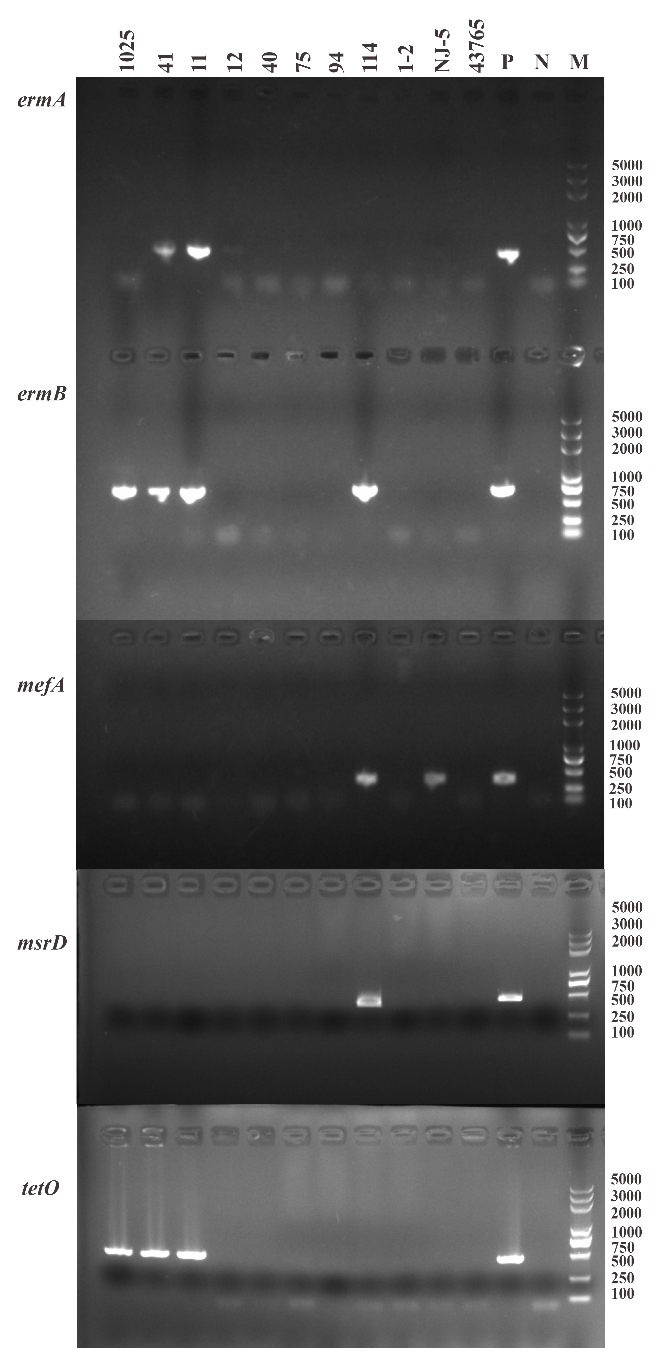

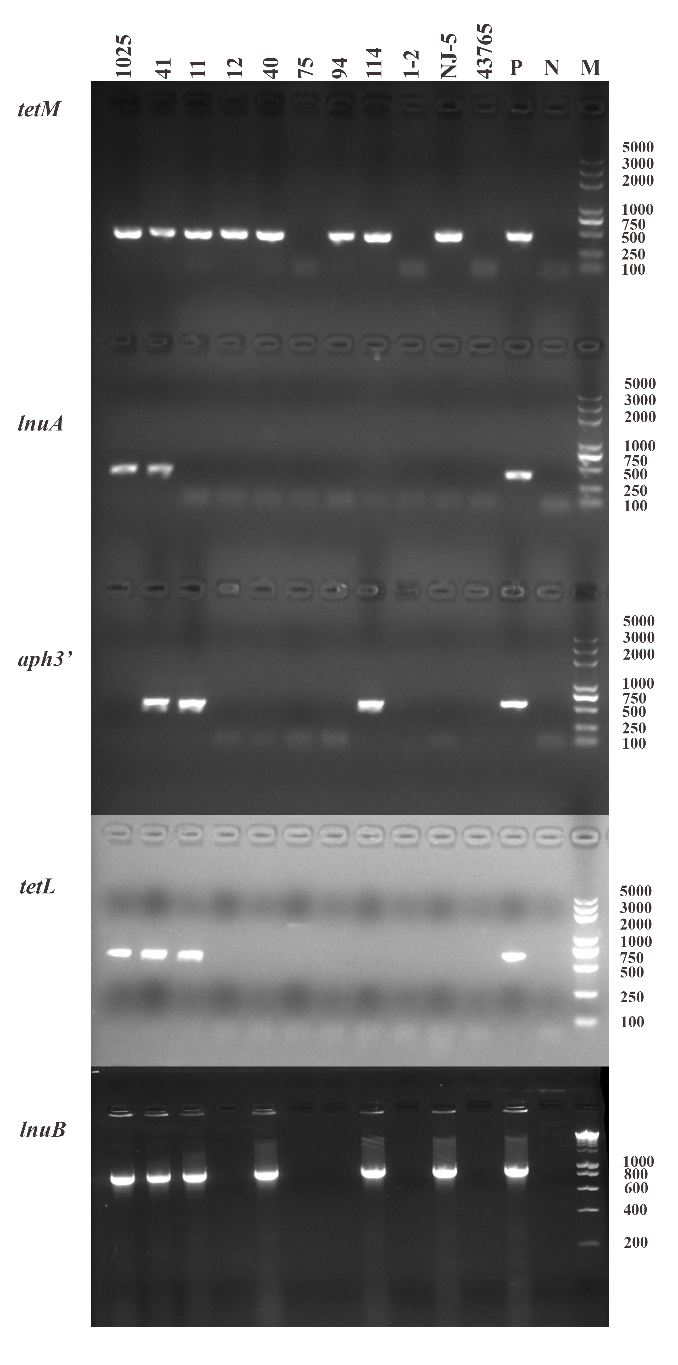


**References**

Bozdogan, B., Berrezouga, L., Kou, M. S., Yurek, D. A., Farley, K. A., Stockman, B. J., et al. (1999). A new resistance gene, linB, conferring resistance to lincosamides by nucleotidylation in Enterococcus faecium HM1025. *Antimicrob. Agents Chemother.* 43, 925–929.

Lina, G., Quaglia, A., Reverdy, M. E., Leclercq, R., Vandenesch, F., and Etienne, J. (1999). Distribution of genes encoding resistance to macrolides, lincosamides, and streptogramins among staphylococci. *Antimicrob. Agents Chemother.* 43, 1062–1066.

Lüthje, P., and Schwarz, S. (2007). Molecular basis of resistance to macrolides and lincosamides among staphylococci and streptococci from various animal sources collected in the resistance monitoring program BfT-GermVet. *Int. J. Antimicrob. Agents* 29, 528–535. doi:10.1016/j.ijantimicag.2006.12.016.

Malhotra-Kumar, S., Lammens, C., Piessens, J., and Goossens, H. (2005). Multiplex PCR for simultaneous detection of macrolide and tetracycline resistance determinants in streptococci. *Antimicrob Agents Chemother* 49, 4798–4800. doi:10.1128/AAC.49.11.4798-4800.2005.

Sutcliffe, J., Grebe, T., Tait-Kamradt, A., and Wondrack, L. (1996). Detection of erythromycin-resistant determinants by PCR. *Antimicrob. Agents Chemother.* 40, 2562–2566.

Tait-Kamradt, A., Davies, T., Cronan, M., Jacobs, M. R., Appelbaum, P. C., and Sutcliffe, J. (2000). Mutations in 23S rRNA and Ribosomal Protein L4 Account for Resistance in Pneumococcal Strains Selected In Vitro by Macrolide Passage Mutations in 23S rRNA and Ribosomal Protein L4 Account for Resistance in Pneumococcal Strains Selected In Vitro by Macroli. *Antimicrob. Agents Chemother.* 44, 2118–2125. doi:10.1128/AAC.44.8.2118-2125.2000.Updated.

Warsa, U. C., Nonoyama, M., Ida, T., Okamoto, R., Okubo, T., Shimauchi, C., et al. (1996). Detection of tet(K) and tet(M) in Staphylococcus aureus of Asian countries by the polymerase chain reaction. *J. Antibiot. (Tokyo).* 49, 1127–1132. doi:10.7164/antibiotics.49.1127.

Wisselink, H. J., Reek, F. H., Vecht, U., Stockhofe-Zurwieden, N., Smits, M. A., and Smith, H. E. (1999). Detection of virulent strains of Streptococcus suis type 2 and highly virulent strains of Streptococcus suis type 1 in tonsillar specimens of pigs by PCR. *Vet. Microbiol.* 67, 143–157. doi:10.1016/S0378-1135(99)00036-X.
